# Nanostructured Lipid Carriers to Enhance the Bioavailability and Solubility of Ranolazine: Statistical Optimization and Pharmacological Evaluations

**DOI:** 10.3390/ph16081151

**Published:** 2023-08-14

**Authors:** Aziz Unnisa, Ananda K. Chettupalli, Reem S. Alazragi, Walla Alelwani, Azzah M. Bannunah, Jameel Barnawi, Padmanabha R. Amarachinta, Suresh B. Jandrajupalli, Badria A. Elamine, Omkalthoum A. Mohamed, Talib Hussain

**Affiliations:** 1 Department of Pharmaceutical Chemistry, College of Pharmacy, University of Ha’il, Ha’il 81442, Saudi Arabia; 2 Department of Pharmaceutical Sciences, Center for Nanomedicine, School of Pharmacy, Anurag 10 University, Venkatapur, Ghatkesar, Medchal, Hyderabad 500088, India; anandphd88@gmail.com (A.K.C.); padnabhaachim@gmail.com (P.R.A.); 3 Department of Biochemistry, College of Science, University of Jeddah, Jeddah 21959, Saudi Arabia; rsalazragi@uj.edu.sa (R.S.A.); welwani@uj.edu.sa (W.A.); 4 Department of Pharmaceutics, College of Pharmacy, Umm Al-Qura University, Makkah 24382, Saudi Arabia; ambannunah@uqu.edu.sa; 5 Department of Medical Lab Technology, Prince Fahd Bin Sultan Research Chair, Faculty of Applied Medical Sciences, University of Tabuk, Tabuk 71491, Saudi Arabia; jbarnawi@ut.edu.sa; 6 Department of Preventive Dental Sciences, College of Dentistry, University of Ha’il, Ha’il 81442, Saudi Arabia; s.jandrajupalli@uoh.edu.sa; 7 Department of Radiology, College of Applied Medical Sciences, University of Ha’il, Ha’il 81442, Saudi Arabia; b.alamin@uoh.edu.sa; 8 Department of Special Education, College of Education, University of Ha’il, Ha’il 81442, Saudi Arabia; o.hamid@uoh.edu.sa; 9 Department of Pharmacology and Toxicology, College of Pharmacy, University of Ha’il, Ha’il 81442, Saudi Arabia; md.talib@uoh.edu.sa

**Keywords:** ranolazine, nanostructured lipid carrier, transdermal, in-vivo absorption, Box-Behnken design, permeation

## Abstract

Chronic stable angina pectoris is the primary indication for ranolazine (RZ), an anti-anginal drug. The drug has an anti-ischemic action that is unaffected by either blood pressure or heart rate. Due to the first-pass effect, the drug has a reduced bioavailability of 35 to 50%. The study emphasized developing a novel transdermal drug delivery system of nanostructured lipid carriers (NLCs) for delivering RZ. Many pharmaceutical companies employ lipid nanoparticles as biocompatible carriers for medicinal, cosmetic, and biochemical uses. These carriers are appropriate for many applications, such as topical, transdermal, parenteral, pulmonary, and oral administration, because of the large variety of lipids and surfactants that are readily available for manufacturing. RZ NLCs were made using high-pressure homogenization. Statistical analysis was utilized to find the best formula by varying the concentrations of Precirol ATO 5 (X1), oleic acid (X2), and Tween 80 (X3). Variables such as entrapment effectiveness (EE) (Y1), particle size (Y2), polydispersity index (PDI) (Y3), and zeta potential (Y4) were tested. A variety of tests were performed on the new formulation to ascertain how well it would be absorbed in the body. These tests included in vivo absorption studies, skin permeability assessments, in vitro drug release assessments, and physicochemical analyses. The particle size of RZ-NLCs was shown to be very small (118.4 ± 5.94 nm), with improved EE (88.39 ± 3.1%) and low ZP and PDI (−41.91 ± 0.38 and 0.118 ± 0.028). SEM and TEM analysis confirmed the structure of the NLCs and showed a smooth, spherical surface. Improved RZ-NLCs were used to create NLC gel, which was then tested for elasticity both physically and rheologically. The formulation’s elasticity was investigated. Optimized RZ-NLCs and NLCG were found to have transdermal fluxes of 48.369 g/cm^2^/h and 38.383 g/cm^2^/h, respectively. These results showed that the transdermal delivery of RZ distribution through NLC’s transdermal gel had more significant potential. According to in vivo experiments, the drug’s bioavailability in Wistar rats increased when it was delivered through NLCs. The findings demonstrated that NLCs loaded with RZ successfully transported the RZ to the designated site with no interruptions and that a quadratic connection existed between the independent and dependent variables.

## 1. Introduction

A drug condition known as angina pectoris develops when the heart obtains less oxygenated blood [1]. Potassium channel openers remain an important therapeutic class for angina pectoris and hypertension [2]. Angina pectoris is a chronic illness that affects many people and is linked with significant morbidity and mortality. It often results from heart muscle ischemia brought on by coronary artery spasm or occlusion [3]. Nitrates, calcium channel blockers, beta-blockers, and other anti-anginal drugs treating angina pectoris relieve symptoms and control heart rate [4,5].

Ranolazine (RZ), a piperazine acetamide derivative, acts as an anti-anginal drug. It works by partially inhibiting fatty acid oxidase, which boosts the myocardium’s ability to produce adenosine triphosphate from glucose. As a result, it has anti-ischemic effects that are not dependent on hemodynamic factors like blood pressure or heart rate. The issues above and other co-morbidities will not substantially impact its efficacy. This benefit makes it sound like an efficient anti-ischemic or anti-anginal drug for treating myocardial infarction, cardiac arrhythmias, and unstable chronic angina pectoris [6,7].

The Food and Drug Administration (FDA) approved RZ for angina pectoris therapy in 2006. It is a unique anti-anginal drug with anti-ischemic and metabolic actions. Biopharmaceutical classification places RZ as a class II agent. The first-pass effect on RZ is enormous and erratic. Half-lives of RZ range from 1.4 to 1.9 h, and doses of 500 to 1000 mg twice a day are efficacious [8,9], but the t1/2 of the extended-release (ER) formulation is 7 h. First-pass metabolism, gastrointestinal (GI) side effects, poor absorption, insufficient bioavailability, and the need for a large dosage are all problems with orally administered RZ. Transdermal drug delivery is excellent for treating angina pectoris because of its reduced drug dose, avoidance of the first-pass impact, higher bioavailability, controlled drug administration, and 100% patient compliance. To make RZ more easily administered topically, a nanovesicular drug delivery system composed of nanostructured lipid carriers (NLCs) was created [10].

Many drugs cannot be absorbed via the skin because of the barrier function of the stratum corneum (SC) [11]. Several strategies have been explored to improve transdermal permeability across the SC barrier. The sustained impact that may be achieved with these methods gives them hope for the treatment of chronic illnesses like hypertension [12,13]. The transdermal administration of anti-hypertensive drugs has been extensively researched and developed to overcome the shortcomings of conventional drug delivery methods [14].

Some of the ways that anti-anginal drugs are delivered are through liposomes [15], biodegradable particles [16], micelles [17], dendrimers [18], nanoparticles [19], and nanogels [20]. Lipid-based delivery methods are more biocompatible with skin lipids, making them an attractive carrier for transdermal administration [21]. To create NLCs, solid lipid, liquid lipid, and an aqueous emulsifier solution are combined. Adding liquid lipids to NLCs gives them a crystalline shape comparable to solid lipids, expanding the NLCs’ space for drug storage [22]. Their low cytotoxicity and systemic toxicity come from the physiological and biodegradable lipids they contain, making them ideal for systemic distribution over the skin [23]. A higher surface area is available for skin absorption of the drug in the nano-size range, improving therapeutic effectiveness [24]. A reduction in transepidermal water loss results from the skin sticking to it, forming a thin layer, and exerting an occlusive effect. H_2_O may help expand the SC’s inter-corneocyte spaces, improving drug penetration into deeper layers.

Statistical experimental designs have been popular in recent years for their ability to speed up the creation of novel formulations with fewer trials and better quantify the influence of factors [25]. Response surface methods (RSM) [26] include the central composite design (CCD), the D-optimal design (D-optimal), and the Box-Behnken design (BBD), all of which can help improve different formulations. The three variables used in the experimental design of BBD are located in the central region, the periphery, and the extremes of the process space, respectively. Since there are no hidden areas to explore, doing BBD trials that could involve a significant outlier is a breeze to avoid. In addition, fewer runs are required for BBD than for other models based on the three-level response surface design approach. As a result, BBD has been touted as a tool for improving a wide variety of nanocarriers.

In this study, we used design expert software to create a nanosized lipid carrier that could transport RZ. Different aspects of the optimized NLC formulation were analyzed, including its %EE, particle size (PS), shape, in-vitro release, in-vivo absorption, and skin contact.

## 2. Results and Discussion

### 2.1. Selection of Solid and Liquid Lipids

**Figure 1:**
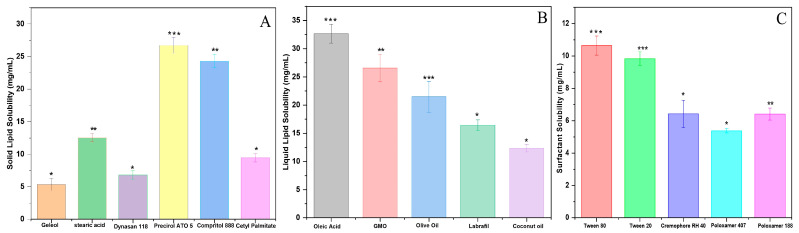
Solubility studies of RZ (**A**) solid lipids, (**B**) liquid lipids, and (**C**) surfactant. Values are expressed in mean ± SD, *n* = 3. * *p* > 0.05, ** *p* > 0.02, *** *p* > 0.001.

The log *p* value of 2.07 ± 0.06 for RZ indicates it is not water-soluble. This drug has high lipophilicity. Figure 1A,B depict the results of studies into the solubility of RZ in solid and liquid lipids, respectively. Precirol ATO 5 was the best option for dissolving the drug, and it was followed by Compritol 888, Stearic Acid, Geleol, Cetyl Palmitate, and Dynasan 118. The oils with the best solubility in water were coconut oil, olive oil, and Labrafil^®^ M2125 CS, followed by oleic acid and glyceryl monooleate (GMO). As a result of this, oleic acid and Precirol ATO 5 were chosen as the solid and liquid lipids in RZ-NLCs.

When testing the miscibility of oil and a hydrophilic filter paper, we observed no droplets when the solid: liquid lipid ratio was 85:15. The anticipated melting temperature of this lipid combination for RZ-NLCs is 58.5 ± 2 °C (solid:liquid: 85:15). The inclusion of a surface-modifying agent, stearyl amine, at a concentration of 0.1% *w*/*v* improved its bioavailability. It has been proposed that stearyl amine be included in the NLC formulation to enhance the interaction of RZ-NLCs with the negatively charged mucosal cells [27]. Positively charged nanoparticle surfaces would be produced by the presence of amine groups.

### 2.2. Selection of Surfactants

Since stable nanoparticles, effective entrapment, and strong lipid-drug interaction were the objectives for the formulation of NLCs, surfactants, including Polysorbate 20, Polysorbate 80, Cremophor RH 40, and Poloxamer 188, were preferred to reduce the solubility of RZ (Figure 1C). In a microscopic analysis, pre-emulsion batches consisting of Poloxamer 407, Poloxamer 188, and Cremophor RH 40 showed moderate sphericity and high to moderate aggregation. Polysorbate 20 was used as a surfactant to achieve reduced accumulation and non-sphericity in the batches. Using polysorbate 80, the resultant particles were round and showed no evidence of aggregation. Polysorbate 80 was employed for further investigations.

### 2.3. Experimental Design Optimization 

Table 1 displays the statistical strategy, which classified the study’s 17 formulations into four variables based on EE, PS, PDI, and ZP. Finding the quadratic coefficients in the second-order polynomial equations for EE, PS, PDI, and ZP helped us understand how the independent and dependent variables are related mathematically. When the R^2^ value was between 0.9437 and 0.9824, a robust connection existed between the polynomial coefficients and the data. In a polynomial equation, a variable with a negative sign suggests an inverse relationship between the component and the response. Optimization is more effective for positive values of the variable in polynomial equations and vice versa. EE, PS, PDI, and ZP were all significantly impacted by each of the three factors (liquid lipid [oleic acid], solid lipid [Precirol], and surfactant [tween 80]). We were able to quantitatively explore the effects of each independent variable and their interactions using an analysis of variance (ANOVA). The Design-Expert^®^ software was used to determine the polynomial coefficient for each dependent variable and to quantify the impact of each independent variable in the development of Equations (1)–(4). We only considered the equations’ most important variables and how they interacted.

#### 2.3.1. Effects of Independent Variables Different Types of Lipids (A): Solid Lipids (B): Liquid Lipids (C): Surfactant on Polystyrene

**Figure 2:**
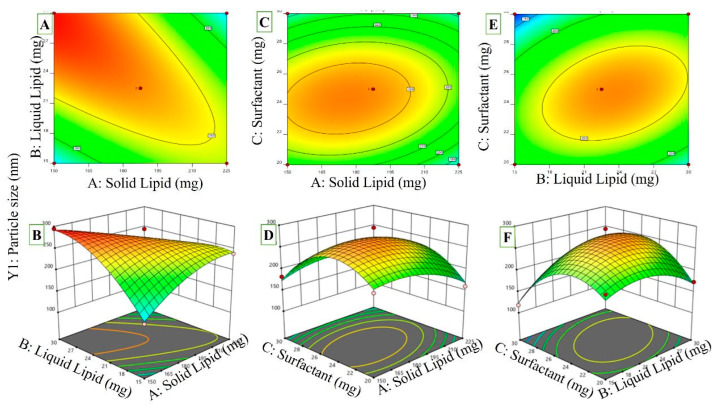
A *p* ≤ 0.05 response surface plot depicting the influence of independent factors and the interaction of dependent variables on the PS of RZ-NLCs ((**A**,**C**,**E**) 2D counter plots for Independent Variables effect on Particle size) ((**B**,**D**,**F**) 3D Response surface plots on particle size).

According to Table 1, PS ranged from a low of 118.4 ± 5.94 nm to a high of 294.8 ± 2.49 nm. PS is a function of the three independent variables shown in three dimensions in Figure 2A–F. PS changes with changes in total lipid concentration in the model’s plot. PS increased with increasing solid lipid to liquid lipid concentrations. More extensive lipid-drug crosslinking yields a giant molecule. As the concentration of lipids rises, more space opens between the bilayers of solid and liquid lipids, allowing for more drug delivery. As the concentration of tween 80 increases, the emulsion droplets shrink, and the interfacial tension between the two phases falls [28]. A steric barrier on the surface of a substance with a high surfactant concentration prevents the smallest particles from adhering to one another [29]. Notable model designations were AB, BC, B^2^, and C^2^. The values for “Predicted R^2^”(0.7498) and “adjusted R^2^” (0.9319) were quite similar (the difference was less than 0.2). The S/N (signal-to-noise) ratio is calculated using Adeq Precision.

PS = +270.34 − 13.13A − 12.03B − 10.75C − 55.25AB + 14.05AC + 29.45BC − 24.60A^2^ − 34.29B^2^ − 60.70C^2^ (1)

According to the PS equation, A, B, and C all have zero influence on PS. The PS of NLCs was shown to be dramatically attenuated by increasing their solid lipid (factor A) content (*p* ≤ 0.05). Similar outcomes were observed when measuring the PS of NLCs before and after a rise in liquid lipid content (B). Since oleic acid and precirol ATO^®^ may reduce system viscosity and surface tension, this may increase the PS of the resultant NLCs. It has been shown [30] that mixing the liquid lipid with the solid lipid while forming a solid lipid nanoparticle (SLN) dispersion may reduce the system’s viscosity. Researchers demonstrated that shifting from a lipid matrix composition containing 15% liquid lipids to one containing 30% successfully decreased particle size. Similar to how a higher concentration of surfactant (C) can quickly emulsify the whole lipid contents of the formulation, producing smaller nanoparticles with a narrower particle size distribution (PS).

Factor interactions (AB, BC, and CA) have a substantial (*p* ≤ 0.05) effect on the NLC’s PS, as shown in Figure 2. The summative effect of two variables (AC and BC) and the multiplicative effect of a third (degree of freedom (DF)) had a significant impact on NLC size (AB interaction). The fact that PS decreased with increasing concentrations of solid and liquid lipids (AB) demonstrated Factor X’s detrimental effects. The AC interaction between the solid lipid and surfactant, which is receiving increasing attention in the NLC formulation, has a significant impact on the size of the particles. Contrarily, at higher concentrations, the interaction between a liquid lipid and a surfactant had a negative effect on particle size (BC interaction).

#### 2.3.2. Effect of Independent Variables on EE 

**Figure 3:**
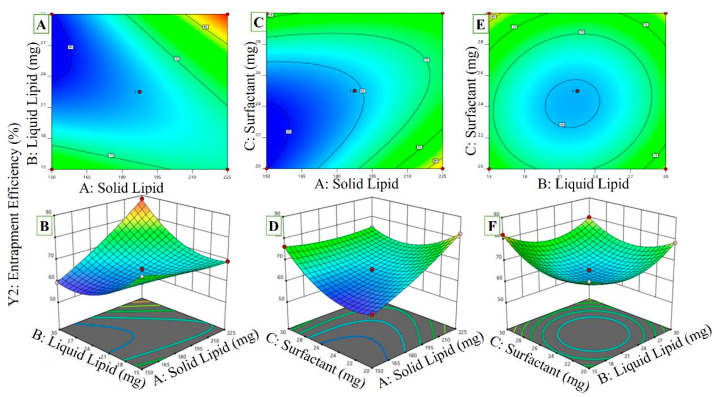
The plot of the response surface shows the substantial (*p* < 0.05) influence of independent factors and interactions between dependent variables of RZ-NLCs ((**A**,**C**,**E**) 2D counter plots for Independent Variables effect on Entrapment Efficiency) ((**B**,**D**,**F**) 3D Response surface plots on entrapment Efficiency).

It is tabulated in Table 1 that EE ranged from a low of 59.37 ± 2.3 to a high of 88.39 ± 3.1%. Three-dimensional plots of the impact of these independent factors on EE are shown in Figure 3A–F (Y1). Precirol has been shown to improve the entrapment efficiency of NLCs. Mixing liquid and solid lipids may have disrupted the crystal arrangement, which enhanced EE and improved drug entrapment efficiency. Raising the surfactant concentration increases EE, and doing so in the external phase might make it easier for the drug to be partitioned out of the internal phase and into the external one. Now that partitioning has been fine-tuned, the drug is more soluble in the external aqueous phase. As a result, more of the drug might be dissolved within that time. The model terms A, C, AB, AC, B^2^, and C^2^ are included. The calculated R^2^ of 0.9503 agreed with the modified R^2^ of 0.9382.

EE = +64.52 + 5.29A + 0.5500B + 2.23C + 8.96AB − 5.98AC − 1.50BC + 1.70A^2^ + 7.11B^2^ + 7.16C^2^ (2)

The %EE of the drug was found to decrease considerably (*p* < 0.05) when the solid lipid content was increased. Contrarily, when surfactant concentration was raised, the drug’s %EE was in the NLCs. A higher surfactant concentration may cause the particle to develop a thicker coating of NLCs on its outside, entrapping some drugs. Zhuang and Das postulate that sufficient surfactant is needed to solubilize drug molecules within the lipid lattice structure and on the outer surface of nanoparticles [31,32].

Reducing the amount of surfactant used and lengthening the homogenization time leads to a significant (*p* ≤ 0.05) rise in the drug %EE in the RZ-NLCs. In contrast, the proportion of effective drugs increased due to the interaction between solid and liquid lipid concentrations (AB interaction). Increasing the concentration of the solid and liquid lipid components of the RZ-NLCs may improve the drug’s %EE. Since more solid lipids are in the system, more drug molecules can fit, resulting in a higher percentage of the drug’s %EE in the RZ-NLCs. 

#### 2.3.3. Effect of Independent Variables on PDI

**Figure 4:**
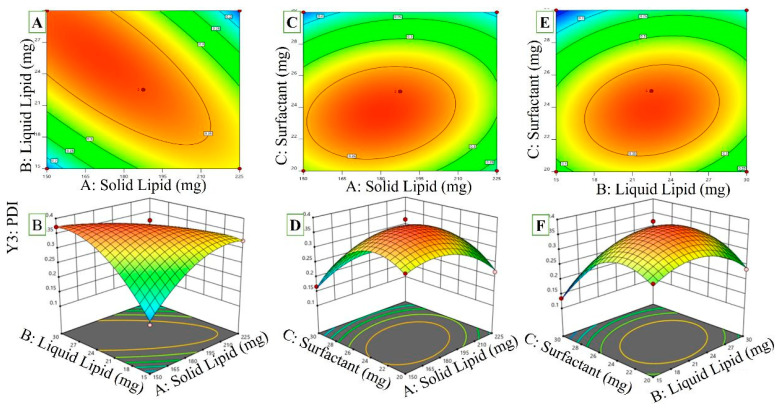
PDI of RZ-NLCs is shown to be significantly (*p* ≤ 0.05) influenced by the liquid lipid concentration, as seen by this response surface plot, including their mutual influences on one another. ((**A**,**C**,**E**) 2D counter plots for Independent Variables effect on PDI) ((**B**,**D**,**F**) 3D Response surface plots on PDI).

The PDI values found to fall in the range of 0.118 ± 0.028 to 0.394 ± 0.043 are shown in Table 1. This three-dimensional graph shows how many variables influence the performance growth index (Figure 4A–F). The PDI is positively correlated with the surfactant amount present and the overall lipid content. As can be seen, the “Adj R^2^” of 0.8713 was quite close to the “Predicted R^2^” of 0.8578. This model may help you navigate the design space, and its precision is expressed as a ratio of signal to noise. In this scenario, all of the individual and combination variables were represented by significant model terms.

PDI = +0.3744 − 0.0144A + 0.0079B − 0.0468C − 0.0930AB + 0.0282AC + 0.0298BC − 0.0562A^2^ − 0.0622B^2^ − 0.0975C^2^ (3)

If you increase the proportion of oleic acid, the PDI will decrease. The NLC particles will be more evenly dispersed, as demonstrated in Equation (3). Earlier studies [33] showed that oleic acid had a similar impact on NLCs. Figure 4 shows the relationship between surfactant and lipid content in a liquid (AC interaction). The PDI was significantly lowered due to this interaction. This suggests that the PDI values would drop with increasing solid and liquid lipid concentrations. The interaction (CD interaction) between the liquid lipid and surfactant concentrations was also shown to be synergistic by the PDI values. Consequently, the PDI values would rise, and the RZ-NLC particles would be less uniform if the liquid lipid and surfactant concentrations were increased concurrently. Above-optimal concentrations may cause surfactant deposition on the nanoparticles’ outer surfaces, raising the PDI values. Similarly, the PDI values of NLCs increased as the surfactant content increased [34].

#### 2.3.4. Effects of Variables on ZP

The RZ-NLC formulations’ negative charge was achieved by the ionization of fatty acids (such as glyceryl palmitostearate, which may be found in oleic acid, and Precirol ATO^®^) and dextran sulfate residue. As seen from Equation (4), increasing the quantity of oleic acid had a detrimental effect on the nanoparticles, leading to a more significant negative charge and, hence, a higher ZP of RZ-NLCs. Over-ionization of carboxylic groups in oleic acid (a kind of liquid lipid) might be responsible [35]. Glyceryl palmitostearate, a solid lipid, similarly reduced ZP values; increasing glyceryl palmitostearate concentration decreased the ZP of nanoparticles. Glyceryl palmitostearate’s carboxylic acid contributes to a more significant accumulation of negative charges on the nanoparticles’ surface.

ZP = −30.06 − 0.8000A − 0.0625B − 4.21C − 1.33AB + 2.63AC − 0.3000BC − 3.55A^2^ − 2.62B^2^ − 2.42C^2^ (4)

**Figure 5:**
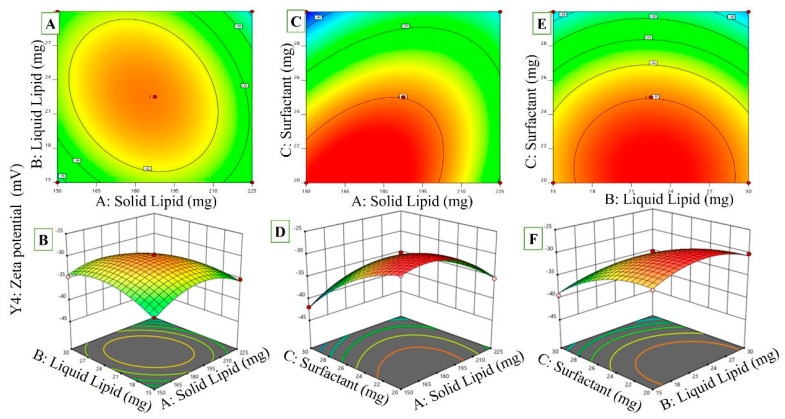
The response surface plot revealed that the ZP of RZ-NLCs was strongly influenced by the liquid lipid and surfactant concentrations (*p* ≤ 0.05), as well as by the interactions between BC, AB, and AC. ((**A**,**C**,**E**) 2D counter plots for Independent Variables effect on Zeta potential) ((**B**,**D**,**F**) 3D Response surface plots on zeta potential).

Considerably beneficial effects of the AC interaction on NLCs’ ZP and significantly adverse effects of the CA interaction were observed (Figure 5). By raising the solid lipid content and lowering the surfactant concentration, the negative charge on the nanoparticles may be decreased, and ZP values can be lowered. The ZP for RZ-NLCs is reduced because a lower surfactant concentration cannot ionize the whole quantity of solid lipid, resulting in a lower negative charge on the nanoparticles’ surfaces. Further, the ZP value of RZ-NLCs would rise dramatically if the concentrations of liquid lipids and surfactants were raised simultaneously (BC interaction). A higher surfactant concentration in the formulation would result in a more significant ionization of the carboxylic acid. 

### 2.4. Point Prediction 

**Figure 6:**
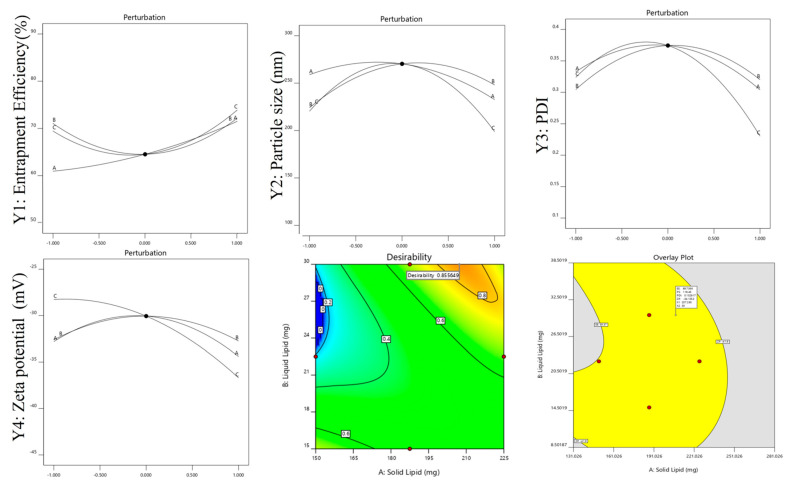
The perturbation of dependent variables, desirability, and overlay plot of optimized formulation.

By changing the amounts of liquid lipid (30 mg oleic acid), solid lipid (207.2 mg GMS), and surfactant (20 mg tween80), the best formulation (RZ-NLCs) was made. Particle size (118.4 ± 5.94 nm and 117.438 nm), encapsulation efficiency (88.39 ± 3.1 and 89.946), and PDI (0.118 ± 0.028 and 0.150) were all found to be within a small margin of error of the experimentally determined value of the RZ-NLCs optimized formulation. The validity of the anticipated importance of replies was found to be 98.23%, 99.51%, and 100.08%, respectively, proving that the optimal formulation was selected. Based on the perturbation plots and desirability plots, the optimized formulation was selected (Figure 6). The ANOVA analysis confirmed the significance of all model variables, including those responsible for interaction effects. This conclusion was reached as a result of the fact that the model variables were significant. Following further optimization via point prediction, the RZ-NLC was verified by applying carbopol as the gelling agent before being evaluated for additional parameters. The optimized formulation is shown in Table 2 with predicted and experimental values. 

### 2.5. Effects on Particle Size, PDI, and ZP

**Figure 7:**
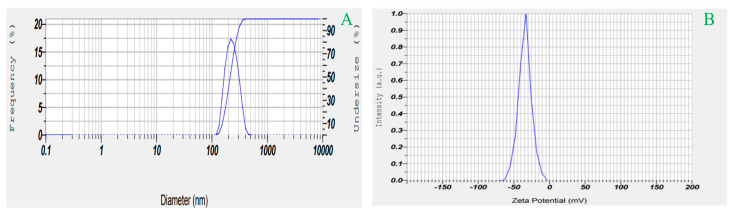
Particle size distribution and Zeta potential of optimized RZ-NLC formulation (**A**) Particel size distribution (**B**) Zeta potential.

Vesicle diameters for the prepared RZ-NLCs ranged from 118.4 ± 5.94 (F10) to 294.8 ± 2.49 (F17). The PDI was measured to be 0.168 ± 0.033, below the cutoff value of 0.5 and suggesting a normal distribution (Figure 7). When the concentration of liquid lipids was raised from 15 to 30 mg, solid lipids were kept at 187.5 mg, and the size and PDI of the formulations shrank while the ZP rose. Perhaps this is because there is less solid lipid to help direct the drug. The ZP decreased and the size altered when the quantity of solid lipids was raised from 150 to 225 mg while the amount of liquid lipids was held constant. The NLCs grew in size when solid lipid content increased.

Extremely negative zeta potential values (−29.6 ± 1.26 mV) were measured, showing that the produced RZ-NLCs were very stable and that the vesicles had not coagulated or aggregated. Strong evidence of the RZ’s orientation in the lipid metric was found to be an opposing surface charge in RZ-NLC compositions. The surface charge is essential if you wish to create a stable colloidal preparation. It is a surrogate for NLCs’ physical stability and may influence their environmental and cellular fates. A ZP of 30 mV is necessary for the electrostatic stability of a nanoformulation. In contrast, a ZP of 20 mV is optimal for the combined stabilization of electrostatic and steric effects. The research was conducted by a group of scientists [36].

### 2.6. Measurement of EE and Drug Concentration

The drug concentration of NLC formulations was between 96.89 ± 0.18 and 98.97 ± 0.20 mg. The calculated %EE for the NLC formulations was between 59.37 ± 2.3 and 88.39 ± 3.1%. Considering RZ is very lipophilic, it was discovered that %EE contains a lot of lipid nanoparticles (Table 1). Long-chain fatty acids covalently bound to the glyceride may be to blame for this enhanced capacity to accommodate lipophilic drugs. Liquid lipid, which has a less structured lipid matrix, leads to defects that generate vacuum regions where drug molecules may get stuck [37]. The drug was dissolved in molten lipids at temperatures higher than the threshold at which solid lipids could melt. Neither the drug nor its metabolites leaked or precipitated throughout the manufacturing process. The solubility of the drug in the lipid phases, the nanoparticle crystallinity index and, by extension, the diffusion coefficient of the drug, the manufacturing method, and the circumstances utilized all have an impact on the release properties of NLCs, which in turn are impacted by the EE. The lipidic nanoparticles in NLCs immobilize the administered drug by remaining in a solid form, which increases EE. Encapsulating a substantial dosage of the medicine in lipid nanoparticles and having it delivered via the lymphatic system is one way to avoid first-pass metabolism.

### 2.7. Characterization of Optimized RZ-NLCG

#### pH, Viscosity, Spreadability, and Drug Content Estimation

When applied topically to the skin, the optimized RZ-NLCs-based topical gel had a pH of 6.35 ± 0.26. 38513 ± 0.59 cps and 16.84 ± 0.32 g-cm/s were calculated for consistency and dispersibility, respectively. RZ = 98.37 + 0.23% is the figure derived for the number of drugs found. The created topical gel was found to have a shear thinning property (pseudoplastic flow) and a viscosity of 35,000–40,000 cps, making it ideal for topical use, according to rheological experiments. Several aspects of textural examination were considered, including stability, reliability, hardness, cohesion, and viscosity index. Hardness is 0.158 ± 0.01, toughness is 0.492 ± 0.15, consistency is 1.486 ± 0.23, cohesiveness is −0.08 ± 0.58, and the index of viscosity is −1.068 ± 0.21. Below is Table 3, including the assessment parameters used to assess the enhanced RZ-physicochemical NLCGs and mechanical properties.

### 2.8. Solid-State Characterization

#### 2.8.1. Drug-Excipient Compatibility Studies by DSC

**Figure 8:**
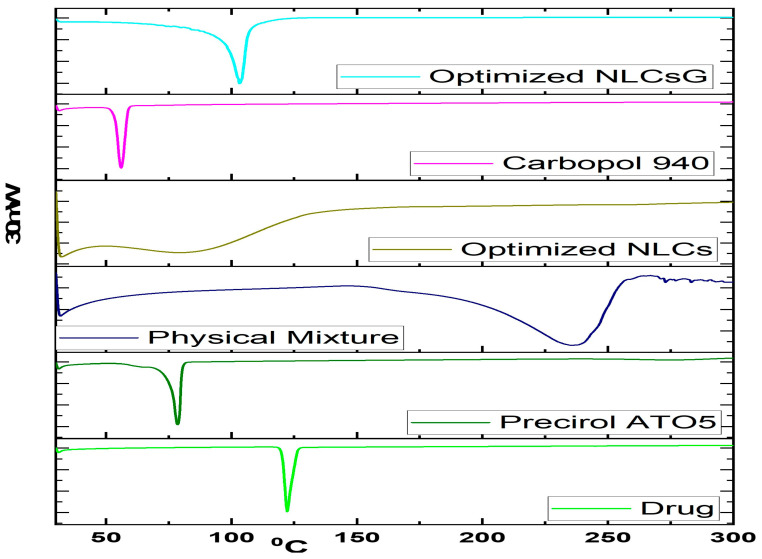
DSC thermal examination of (pure RZ, Precirol ATO 5, a physical mixture of RZ, Oleic acid, and tween 80 (1:1), optimized RZ-NLCs, Carbopol-940, and optimal RZ-NLCG.

The lipids in NLCs had the lowest melting point of any pure drug, lipid, RZ-NLC, Carbopol-940, or RZ-NLCG formulation. In the DSC thermogram of pure RZ, the endothermic peak at 166.4 °C and the 545.84 J/g enthalpies show that the material is entirely crystalline. For the pure substance, an endothermic peak was recorded at 64.38 °C. When the drug was combined with oleic acid and tween 80, the calculated enthalpy values were 173.7 J/g, with the drug’s peak temperature being 0 °C and the lipids’ peak temperature being 72.86 °C (Figure 8). The percentage of crystals decreased to 89.34% as a consequence. The drug’s melting endotherm was maintained with a slight shift due to temperature. Both the peak shape and enthalpy may be affected by the drug-to-excipient ratio. Small deviations in the melting endotherm of the medicine may not always indicate incompatibility since the combination of the drug and the excipient can affect the purity of either component. In RZ-NLC and RZ-NLCG formulations, the drug is converted from its crystalline state to an amorphous form, as shown by the disappearance of the endotherm peak. The crystallinity of NLCs was estimated at 23.64%, whereas that of NLCG was measured at 29.28%. 

#### 2.8.2. Powder X-ray Diffractometry

**Figure 9:**
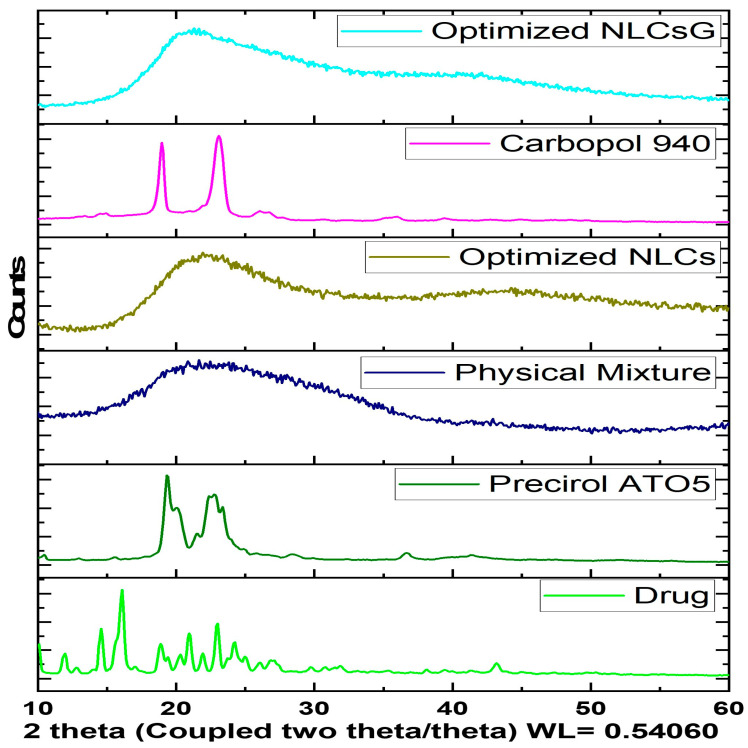
The X-ray diffraction patterns for pure RZ, Precirol ATO 5, a 1:1 mixture of RZ, oleic acid, and tween 80 optimized RZ-NLCs Carbopol 940, and optimal RZ-NLCG.

Figure 9 shows the PXRD analyses of the optimized RZ-NLCs formulation, the pure drug, the pure lipid, the physical drug mixtures, the lipids, and the surfactant at a 1:1 ratio. Powder X-ray diffraction (PXRD) showed that pure RZ was crystalline because it had clear peaks at 3.34°, 10.79°, 18.61°, 21.85°, 24.26°, 25.50°, 31.57°, and 42.29°. The drug peaks were almost eliminated from the adjusted RZ-NLCs sample. In contrast, the RZ peaks were kept at a lower intensity. This result was no surprise after confirming the drug was not crystalline using RZ-NLCs and RZ-NLCG. There was a reduction in both the overall and lipid peak intensities in the optimized sample. The lower crystallinity of the lipids explains the diminished intensity. The degree of crystallinity between the lipid and drug determines how quickly RZ is released from nanoparticles. DSC examination of the samples confirmed this loss of crystallinity.

#### 2.8.3. Morphology of NLCs Using Scanning Electron Microscopy

**Figure 10:**
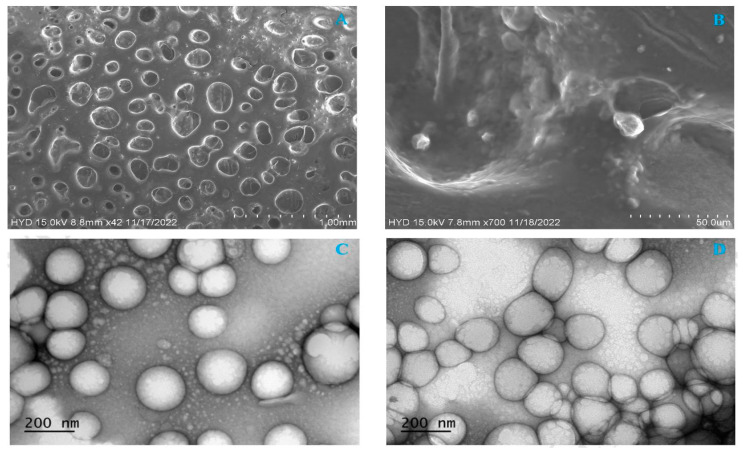
SEM images of (**A**) optimized RZ-NLCs (**B**) Optimized RZ-NLCG; TEM images of (**C**) optimized RZ-NLCs (**D**) Optimized RZ-NLCG.

Scanning electron microscopy analysis of RZ-NLC and RZ-NLCG surfaces is shown in Figure 10. Micrographs of the particles show that their increased size and polydispersity due to the sonication have led to an incredible agglomeration phenomenon. The drug-loaded NLC formulations were smooth on the surface. They were evenly distributed throughout, according to the SEM findings (Figure 10). The TEM images of RZ-NLC and RZ-NLCG revealed that NPs were spherical in nature, discrete, and nonaggregate. The TEM image showed that the RZ-NLC and RZ-NLCG particles had a size range between 150 and 200 nm and were spherical. The measurement made with Zetasizer (photon correlation spectroscopy) only estimates the nanoparticles’ size based on the intensity and scattered light. TEM examination is helpful to verify the outcomes of photon correlation spectroscopy and learn more about the morphology of RZ-NLC and RZ-NLCG. According to the TEM image, the developed RZ-NLC and RZ-NLCG had smooth surfaces and no surface drug crystals.

### 2.9. In Vitro Drug Release 

**Figure 11:**
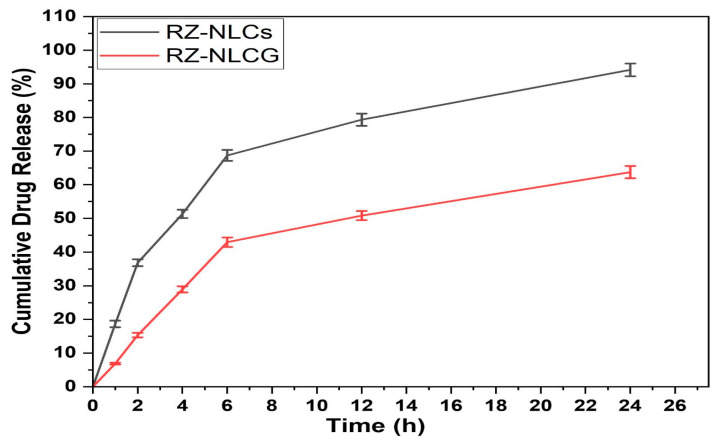
Comparative in vitro drug release profiles of RZ-NLC and RZ-NLCG. Values are expressed in mean ± SD, n = 3.

The optimized RZ-NLC (94.17%) and RZ-NLCG (68.31%) exhibit different drug release profiles, as shown in Figure 11. The enhanced RZ-NLCG displayed a biphasic release pattern with a rapid initial and sustained 24-h release. Although Carbopol forms a thick gel matrix structure, the release of RZ is delayed for many hours after it is dispersed. Significant difficulties arise when treating patients with drugs entrapped in a gel matrix for long periods of time. This release feature helps achieve the target, which is necessary for transdermal drug administration to be effective [38]. Many other kinetic models were tried to explain the drug release data. Still, the Higuchi model was determined to be the most appropriate (0.981). The n value of 0.396 obtained from the Peppas model suggests that drug release was regulated by Fickian diffusion.

### 2.10. Ex Vivo Permeation Studies

**Figure 12:**
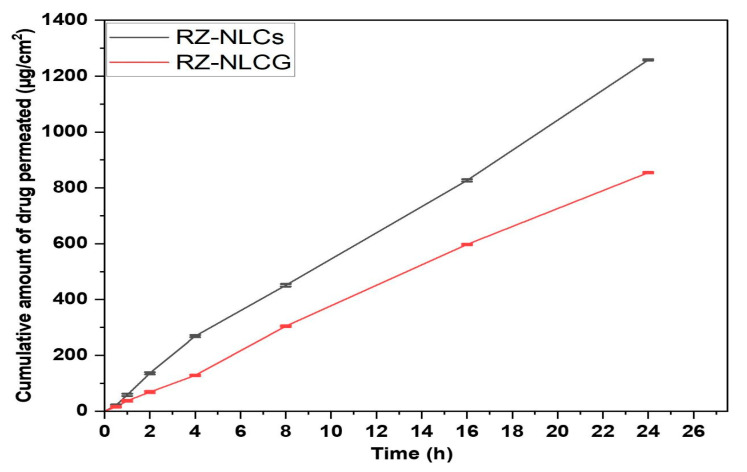
Skin permeation of RZ-NLC and RZ-NLCG into the rat skin. Values are expressed in mean ± SD, n = 3.

Rat skin penetration of RZ-NLCs and the RZ-NLCG formulation was studied using Franz diffusion cells. The rate of RZ penetration per unit area was calculated for the whole surgically removed section of rat skin (Figure 12). The time-dependent permeation curves for the drug showed behavior consistent with typical penetration. Slope analysis of the linear area allowed us to calculate the steady-state flux value, as shown in Figure. The improved RZ-NLC produced 48.369 g/cm^2^/h of RZ flux, much higher than the 38.383 g/cm^2^/h produced by the RZ-NLCG. As dermal retention is crucial in formulating RZ nanoparticles, 1% Carbopol gel was used to disperse the lipid carriers. Therefore, the homogeneity, viscosity, pH, and drug concentration of the synthesized NLCG were analyzed to see how these factors would affect the transdermal skin permeability rate and the aesthetic value of the formulation. Transdermal delivery was shown to be most effective in these settings. The created gel increased in vitro penetration into rat skin and had a high flux to transport RZ to therapeutic concentrations in the biological system.

### 2.11. Pharmacokinetic Study

Figure 12 shows the plasma concentration profiles of RZ in Wistar rats after they were given optimized RZ-NLCG and Ranozex, a commercially available tablet. The Cmax and Tmax of Ranozex Tab were 1039.68 ± 5.48 ng/mL and 2.05 h, respectively, whereas those of RZ-Cmax NLCG were 986.52 ± 8.45 ng/mL and 4.09 ± 0.48 h. 

**Figure 13:**
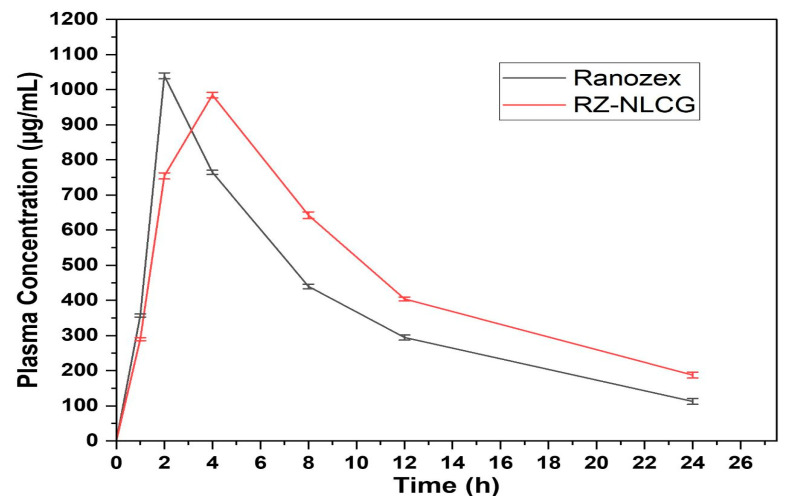
Comparative in-vivo absorption image of (A) RZ-NLCG and (B) Ranozex. Table Values are expressed in mean ± SD, n = 3.

The transdermal gel formulation’s lower Cmax and longer Tmax may be attributed to the stratum corneum’s role as a barrier in this delivery method. The AUC0-t and AUMC t-values for the RZ-NLCG optimized formulation were 702.3659608 ± 14.52 and 3928.505246 ± 18.94 g.h/mL, respectively. While AUC0-t and AUMC t-∞ following oral Ranozex Tab treatment were 779.585782 ± 13.24 and 1437.72977 ± 10.34 µg.h/mL, respectively (Table 4). According to the pharmacokinetic analysis, the NLCG gel formulation of RZ is absorbed more thoroughly than the commercially available tablet. Compared to oral dosing, the substantially higher AUC value seen with RZ-NLCG suggested greater bioavailability of the RZ (Figure 13). Higher absorption was seen with the NLCs-based gel, which may result from the formulation’s effective permeability enhancers (surfactant and lipid). Additionally, the RZ-NLCG formulation may have achieved higher bioavailability (1.64 times) than the oral formulation since it bypassed the liver’s first-pass metabolism.

### 2.12. In Vivo Pharmacodynamic Studies

#### 2.12.1. DOCA Salt Model for Hypertension

**Figure 14:**
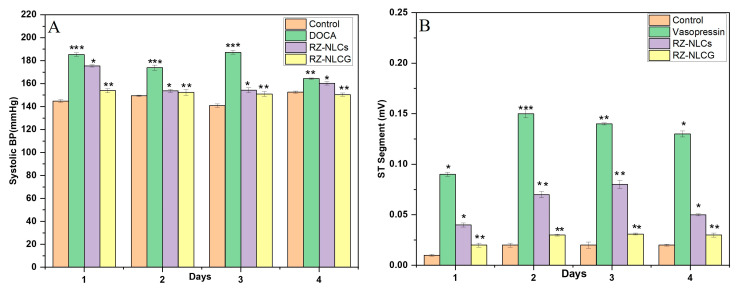
(**A**) Effect of RZ-loaded NLCsG on increase in systolic blood pressure in comparison to that of NLCs on days 1 to 4. Values expressed as mean ± SD. (**B**). Effect of RZ-loaded NLCsG on ST segment normalization on vasopressin-induced ST segment elevation in comparison to that of NLCs on days 1 to 4. Values expressed as mean ± SD, n = 3. * p > 0.05, ** p > 0.02, *** *p* > 0.001.

When animals were treated with DOCA salt, the blood pressure significantly increased after 28 days, indicating hypertension induction. The effect of RZ-NLCs and RZ-NLCG on the systolic pressure for up to 4 days is shown in Figure 14A,B. It was seen from the figure that the blood pressure reached the expected value (150 mm Hg) after administration of RZ-NLCs on the second day. However, the RZ-NLCs could sufficiently control blood pressure thereafter. RZ-NLCG maintained the blood pressure in the normal range (150 mm Hg) for up to 3 days, indicating their ability to sustain the drug release and maintain therapeutic drug concentration for a prolonged period.

#### 2.12.2. Vasopressin Model for Angina

Vasopressin causes the small coronary artery to narrow and raises overall coronary resistance. Following the administration of vasopressin, the ECG alterations (ST-segment elevation) were thought to show the existence of myocardial ischemia, which may be caused by coronary vasoconstriction [21]. The ECG would return to normal after the injection of a coronary vasodilator, which would lessen coronary constriction. The established coronary vasodilator effect of RZ would restore a normal ECG. In a typical ECG, the ST segment measured 0.0142 mV. The vasoconstrictor effect of vasopressin caused the ST segment to rise to 0.1203 mV after being intravenously administered (1 IU/kg). As observed in Figure 12 [ECG 3(A, B, C)], the RZ-NLCs could not sustain an effective drug concentration over an extended period since they failed to normalize the ST segment elevation after the first day appreciably. ECG 4 (A, B, C, and D) demonstrates that RZ-NLCG successfully maintained the impact of normalization of the high ST segment for three days in a row. Figure 13B depicts the outcome of topically applying NLC gel before delivering vasopressin. The vasopressin-induced ST segment elevation was normalized by NLCs gel for up to 3 days, and there was no discernible difference in the ST segment on any of the 3 days (*p* ≤ 0.05). This shows that the coronary vasodilator effects of RZ-loaded NLCsG were present for a considerable time.

#### 2.12.3. Histopathological Analysis 

**Figure 15:**
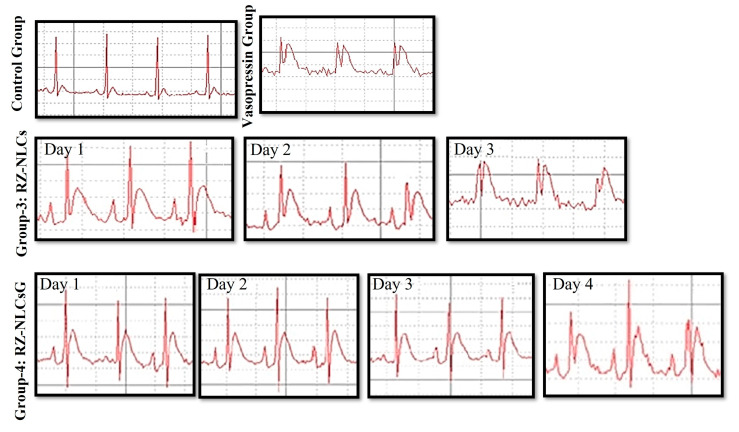
ECG Changes showing the ST segment normalization of (1) control group, (2) vasopressin control group after i.v. administration of vasopressin, (3) group after RZ-NLCs administration on days 1, 2, and 3, and (4) group after RZ-NLCsG administration on days 1, 2, 3, and 4.

**Figure 16:**
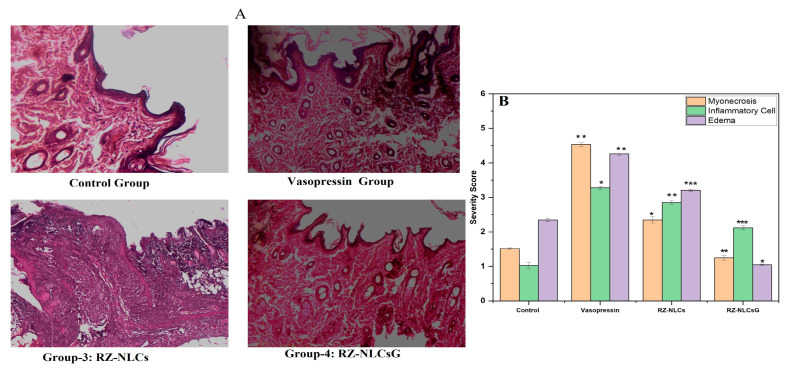
(**A**) Histopathological alterations are affected (10×) by RZ-NLCs and RZ-NLCsG. Normal architecture is seen in the vehicle-treated group, but myonecrosis, inflammation, and edema are seen in the vasopressin-control group. When compared to those treated with the RZ-NLCsG group, it exhibits considerable skin protection by reducing myonecrosis, edema, and inflammation. (**B**) Severity score for blinded histological evaluation in comparison to RZ-NLCsG-treated group. Values are expressed in mean ± SD, n = 3 * p > 0.05, ** p > 0.02, *** p > 0.001.

The effects of RZ-NLCs and RZ-NLCsG on the histopathological changes in skin tissue in rats treated with vehicle and drug are shown in Table 5. The light micrograph of skin exposed to a vehicle in Figure 15 shows the usual architecture without any fraying or infarction. The light micrograph of the vasopressin-control group reveals red blood cell extravasation, myophagocytosis, edema, inflammatory cell infiltration, and localized confluent necrosis of muscle fibers. (Figure 16A). A small amount of edema and a significant decrease in infarction are shown in a rat’s skin given RZ-NLCs and RZ-NLCsG treatment, showing standard myocardial architecture. Blinded histological evaluation using severity grading produced a similar outcome. In the vasopressin-control group, extensive skin tissue damage was discovered (Figure 16B). (Myonecrosis, Inflammatory cells, Emphysema). The group that received NLCsG treatment was shown to have the most minor damage overall, demonstrating the efficiency of the cardioprotective.

### 2.13. Stability Study

RZ-NLCs with the improved formulation were stable for 6 months when kept at 5 ± 3 °C (refrigerated) (Table 6). Six months of stability testing at 5 ± 3 °C showed no statistically significant changes in PS, PDI, ZP, or %EE. The improved formulation, however, degraded after being held for a month at 25 ± 2 °C, 40 ± 2 °C/75 ± 5% RH. PS and PDI were significantly increased, but EE decreased considerably. After just one month of stability testing, the samples were useless for measurement due to nanoparticle aggregation and stickiness.

## 3. Materials and Methods

Dr. Reddy’s Laboratories in Hyderabad, Telangana, generously provided a sample of RZ. We ordered the methanol and HPLC-grade liquid from Merck in Mumbai, India. From “Gattefosse India Pvt. Ltd. (Mumbai, India)”, we ordered Glyceryl monostearate (GMS), “Labrafil M 2125, Peceol” (oil), and Labrafil M 1944. Every other chemical and solvent used were of analytical purity and purchased only from reliable sources. SD Good Compounds (Mumbai, India) supplied Tween-80 and Carbopol 940.

### 3.1. Selection of Lipids (Solid and Liquid)

The effects of various lipids on the solubility of the drug (RZ) (solid and liquid) All lipids (solid or liquid) were weighed to the nearest gram and then placed in amber-colored glass vials. When the RZ was raised and the lipids were melted in at a concentration of 2 mg/mL, the result was a much-improved product. Twenty-four hours were spent incubating the RZ/lipids combination at 100 rpm and 10 °C above the solid wax’s melting point [39,40].

### 3.2. Collection of Solid-Liquid Lipid Ratio/Miscibility Study 

Different combinations of liquid and solid sterols (50:50, 40:60, 30:70, 20:80, 15:85, 10:90, and 5:95) were tested for their miscibility. One gram of lipids was carefully measured, then melted at a temperature slightly above the solid lipid melting point in an incubator with a water bath stirred at 100 revolutions per minute for about an hour. By spreading the cooled, solidified melt over filter paper and visually analyzing it for oil streaks or droplets, the researchers could determine whether or not the lipids were miscible in both the solid and liquid states (if any). After further developing RZ-NLCs [41] using a lipid mixture, it was shown that neither oil staining nor droplet formation occurred at temperatures higher than 40 °C.

### 3.3. Development of RZ-NLCs

The RZ-NLCs were created using the HPH (high-pressure homogenization) method. Briefly, we added RZ to a mixture of Precirol^®^ ATO 5, stearyl amine, oleic acid, and Phospholipon^®^ 90H that had been heated to a temperature of 10 °C over the soft argument of each lipid. Aqueous surfactant solutions (conductivity 0.058 S/cm) were obtained from triple-distilled water. The lipid mixture and the heated aqueous surfactant solution were blended using a “high-shear homogenizer” (IKA^®^ T-25 digital Ultra-Turrax^®^, IKA-Werke, Staufen im Breisgau, Germany) at a speed of 13,000 rpm. The lipid liquid was heated to the same level as the surfactant solution in water. The RZ-NLCs were made by creating a pre-emulsion and then homogenizing the mixture under high pressure. The drug-loaded NLCs were cryoprotected with mannitol before being freeze-dried at −80 °C [27]. The lyophilization process took approximately an hour and thirty minutes at room temperature (25 °C). In contrast, the secondary drying took four hours at zero degrees Celsius. A final moisture content of around 0.2% was achieved via lyophilization at 1 Pa pressure and a −70 °C cold trap temperature.

### 3.4. Statistical Optimization of the RZ-NLCs

Producing pilot lots allowed us to test out different chemical compositions and dial in the processing variables that would ultimately impact RZ-NLCs’ growth potential. The total amount of solid-liquid lipids, the concentration of surfactant and emulsifier, the rate at which the homogenization process is carried out, and other factors appear to have a significant impact on the final product’s “particle size” (PS), “Polydispersity Index” (PDI), “zeta potential”, and “entrapment efficiency” (EE). Particle size distribution is susceptible to homogenization rate. Other characteristics, such as the amount of lipids and emulsifier concentration, may also impact the PS and EE of drug-loaded NLCs. This being the case, we used a BBD consisting of three phases and three components to create NLCs [42]. Table 7 displays the relationships and differences between the dependent and independent variables. The following equations were derived from the mathematical model, illuminating the relationship between the independent and dependent variables:

Y = B0 + B1X1 + B2X2 + B3X3 + B12X1X2 + B13X1X3 + B23X2X3 + B11X1^2^ + B22X2^2^ + B33X3^2^ eq. (5)

where “X1”, “X2”, and “X3” are independent variables; “B0” stands for the total number of observations; “B1”, “B2”, and “B3” are regression values; and “Yi” is the dependent variable of interest. Response surface plots (RSPs) were generated in “Design Expert Software” version 12.0.3 using the equations (Stat-Ease, Inc., Minneapolis, MN, USA).

The optimal formulations were established using the notion of desire functions. The desirability function aggregates the results into a single metric, allowing more accurate forecasting of the optimal values for the elements under investigation. As a result, it was decided that the optimal formulations should be chosen based on their small PS, high ZP, and high EE. The popularity function controls all replies to the range to optimize the overall measure. Acceptable (most desirable) response values are denoted by the desirability value 1, whereas unsatisfactory values are represented by the desirability value 0.

### 3.5. Characterization of RZ-NLCs

#### 3.5.1. Determination of PS, PDI, and ZP

Dynamic light scattering (DLS) and a “Malvern nano zeta sizer” (Malvern, UK) were used to determine the NLCs’ standard PS (Z-Ave) and polydispersity index (PDI). 1.330 flush value, 1.46 refractive index, and 0.001 phospholipid absorption index. The master (water) was opened with this set of keys. The electrical surface charge of the NLCs was determined using laser Doppler electrophoresis and their ZP data [43]. After dilution with ultrapure water, the program used the Helmholtz-Smoluchowski equation to determine the ZP. The program determines that a temperature of 25.1 degrees Celsius is ideal. Each sample was repeated five times, and the result (SD) and the “mean standard deviation” were displayed. Microparticle absence was verified by laser deflection analysis using a “Malvern Mastersizer 3000E” to control PS (Malvern, UK). A water dispersant with a 1.33-index refractive, 1.4-index particle refractive, and 0.001-index “absorption index” was used to conduct the tests. PS was determined by utilizing the volume distribution data (D50 and D90) to determine the fraction of particles with diameters in this range or less. We calculated the mean and standard deviation from the results of five different trials (n = 5).

#### 3.5.2. Measurement of EE and RZ Content

We could calculate the RZ concentration and EE of the final RZ-NLCs with HPLC. In Centrisort tubes (Sartorius, Gottingen, Germany), the ultrafiltrate was separated by spinning them at 10,000 rpm for 30 min over a filter membrane with a molecular mass cutoff of 20,000 Da [32]. Its EE was calculated using HPLC analysis of the free drug content in the dispersion medium.

$$EE=\frac{Total\;amount\;of\;drug\;\left(W\right)-\;free\;drug}{Total\;amount\;of\;drug}\times 100$$

where W represented the entire amount of drug given, the concentration RZ was calculated by dissolving roughly 0.1 mL of each formulation in a 50:50 methanol: chloroform solution (1:1). The mobile phase allowed the solution to be diluted. The levels of RZ were measured using high-performance liquid chromatography. In terms of the HPLC equipment, we had: Using a Shimadzu SPD-20A UV-visible variable wavelength detector with deuterium lamp, an SPD-20AD solvent delivery system with double reciprocating plunger pump, and a solvent combination of methanol and water, a Merck (250 mm 4.6 mm i.d., 5 m) Reverse Phase C18 analytical column was constructed (60:40). At a peak wavelength of 237 nm, the drug’s concentration was determined. The method was linear from 5 to 100 ng/mL, with an R2 of 0.999. As the results demonstrate, the technique has a limit of detection (LOD) of 2 ng/mL and a limit of quantification (LOQ) of 4 ng/mL.

### 3.6. Formulation of RZ-NLCG

A longer residence time of a transdermal formulation in the epidermis may result in more efficient drug delivery. It was decided that RZ-NLCs would be more effective as gels because of their better formulation, which could be readily rinsed off the skin. The gelling agent used in the RZ-NLCG and the control gel was Carbopol 940 (1% *w*/*v*). Carbopol and distilled water were combined to make the polymer dispersion, which was then left to rest in the dark to swell. Finally, adding 15% *w*/*w* polyethylene glycol and 0.1% chlorocresol while stirring resulted in a uniform suspension. Triethanolamine (TEA) was used to neutralize the gel formulation, and a moderate stirring rate produced the best Carbopol-based gel (control gel). RZ-NLCG was prepared for characterization by adding the individualized formulation of RZ-NLCs to the already-prepared gel while stirring [44].

#### 3.6.1. Evaluation of RZ-NLCG

Different aspects of the prepared RZ-NLCG formulation, including color, homogeneity, consistency, and phase separation, were assessed. The homogeneity was examined visually after the gels had been placed in the container. We observed any aggregates, and their appearance was noted. A digital pH meter (Mettler Tolledo, Japan) and a glass microelectrode were used to determine the pH of the gel after it had settled for one minute.

#### 3.6.2. Viscosity and Spreadability

The viscosity of our RZ-NLCGs was measured using a rheometer (Anton Paar’s MCR101 Rheoplus) in a thermostatically controlled circulating water bath. A gel sample was extracted from RZ-NLCG and confined within a 2 cm-diameter circle drawn on a glass plate to assess the compound’s spreadability. For five minutes, a weight was rested on the top glass plate while the bottom plate was kept in place (n = 3) [45]. The diameter increased as a result of the application of weight. Calculate as follows to find the percentage dispersion by area: Two square centimeters (A1) and the total size (A2) after the spread. In a nutshell, the formula is

% Dispersion by Area = A2 × A1 × 100

#### 3.6.3. Texture Analysis

The prepared gel’s many textural features were evaluated by measuring its stability, adhesiveness, force of adhesion, and gel strength. Its enhanced formulation, RZ-NLCG, was assessed at a constant sample height in glass jars with a 55 mm diameter and a 40 mm height to a fixed sample height of 30 mm. Preventing the trapping of gas bubbles in the material and maintaining the testing surface as flat as possible helped delay the test activation [28]. Compression mode picture analysis was accomplished using a TA.XT2 plus texture analyzer (Stable Micro System, UK). The mechanical properties of the gels were determined by analyzing the force-time curves generated.

### 3.7. Solid-State Characterization

#### 3.7.1. Drug-Excipient Compatibility Studies by DSC

In the US city of New Castle, DE, differential scanning calorimetry (DSC) tests were done on RZ, Precirol, RZ-NLCs, and RZ-NLCG to see how the drugs interact with each other and how the crystallinity changes when the drugs are added to NLCs. It was indium that was first used to calibrate the device. Heating rates of 20 °C/min were used to rapidly increase the temperature of 8 mg of aluminum in each sample from 20 to 300 °C. Dry nitrogen was used as the effluent gas for this procedure [39].

#### 3.7.2. Powder X-ray Diffraction Characterization of Crystallinity (PXRD)

The crystallinity of RZ was evaluated, and diffraction studies were carried out using PXRD. PXRD studies were monitored using materials subjected to “nickel-filtered CuK ὰ radiation” (40 kilovolts, 30 milliamps) and a range of 2° to 70° in 0.5-s increments (step size, 0.045°). Precirol, RZ-NLC, RZ-NLCG, and pure RZ were all employed as samples in the PXRD study [46].

#### 3.7.3. Morphology by SEM

SEM analysis of RZ-NLCs and RZ-NLCG morphologies (Hitachi, Tokyo, Japan) One drop of a nanoparticle formulation consisting of RZ-NLCs and RZ-NLCG diluted 100 times in double-distilled water was added to a sample container and left to dry in the air. The platinum covering was mechanically sputtered on (JFC-1600 Auto Fine Coater, JEOL, Tokyo, Japan). Using a scanning electron microscope, the sample was then accelerated at 15,000 volts and seen at different magnifications. The SEM imaging process often uses a high vacuum [15].

#### 3.7.4. Transmission Electron Microscopy (TEM) Studies 

Using a TEM apparatus (Hitachi 7100S; Hitachi, Tokyo, Japan), the produced RZ-NLCs and RZ-NLCG formulations’ shape and surface appearance were assessed. A drop of the diluted formulation was applied to a copper grid coated with carbon before being stained with an aqueous solution of 2% uranyl acetate and TEM-ed. 

### 3.8. In Vitro Drug Release

The in vitro release test used a diffusion cell outfitted with 20 mL receptor chambers and a 1.5 cm^2^ diffusion surface. Specifically, a pretreatment membrane with a 0.2-mm pore size was used in the study. The receptor compartment was filled with a 9:1 PBS/ethanol solution. In contrast, the donor compartment contained both samples (RZ-NLC and RZ-NLCG, each corresponding to 5 mg of RZ). Researchers investigated the leak at 37 degrees Celsius with constant stirring. At predetermined intervals, the 0.5-mL sample was removed from the sampling port and replaced with a fresh medium. UV spectrophotometer testing used materials that had been filtered and diluted. Higuchi’s matrices, zero-order, first-order, and kinetic models were used to simulate the release data from the inquiry [47,48]. With the help of the appropriate correlation coefficients, the optimal model was chosen. The drug release mechanism was determined by fitting the data to the Korsmeyer-Peppas model and then selecting the release exponent (n) from the slope of the straight line [49].

### 3.9. Skin Permeation Studies

Stomach skin from the rats was put between the donor and receptor compartments of the diffusion booths, with the stratum corneum side facing the donor compartment and the dermis side facing the “receptor compartment” (area 1.5 cm^2^). The donor compartment of the removed skin contained the enhanced RZ-NLC and RZ-NLCG formulations (equivalent to 5 mg of RZ), whereas the receptor compartment had the PBS: ethanol solution (9:1; 10 mL). During the experiment, a magnetic stirrer running at 100 rpm was used to maintain constant mixing in the receptor compartment. At set intervals, 1 mL samples were taken from the receptor compartment and replaced with fresh media. A further dilution was made before the samples were analyzed by high-performance liquid chromatography (HPLC) to establish the drug concentration. The entire amount of medicine that could flood through the crust was strategized as a purpose for the period, and the covering infusion degree (Flux) was designed after the stable state was achieved [50].

### 3.10. Pharmacokinetics Study

In vivo investigations were performed on male and female “Wistar rats” weighing 200 to 250 g. After the “Institutional Animal Ethics Committee of Nalanda College of Pharmacy” approved the study (Approval No. I/IAEC/NCP/011/2022 WR), the animal housing facility provided the fauna. The rats were housed in a 252 °C environment with a relative humidity of 75–80% and a 12-h light/dark cycle. They were given a tablet diet (Lipton, Kolkata, India) to eat. After the procedure, rats were given RZ dosages of 2.5 mg/kg body weight [51]. The animals were fasted overnight before being divided into two groups of six individuals each. Oral administration of 1.25 g of optimized RZ-NLCG was given to rats in Group A; in contrast, those in Group B received oral administration of a commercially available tablet (Ranozex). The rat abdomens were shaved and used to examine the effects of RZ-NLCG on the skin of the abdomen. The tail vein of rats was used to draw the blood samples, which were then placed in “Eppendorf tubes with disodium EDTA” as an anticoagulant and preserved for dissimilar time pauses (0, 1, 2, 4, 8, 12, 24 h). To examine the plasma samples for the presence of drugs using the HPLC method, they were centrifuged at a speed of 5000 rpm for 15 min. The maximum “plasma concentration”, “Cmax”, and the amount of time it takes to spread the supreme plasma focus, Tmax, are two parameters of biopharmaceutical assessment metrics that may be derived directly from a kinetic plot showing “plasma concentration” vs. time. The trapezoid rule stayed in use to estimate the AUC_0–48_ h and AUC_0-inf_ values. The LC-10 ATVP (Shimadzu, Tokyo, Japan) (SPD-10A) is a binary pump and UV detection system HPLC instrument. A 250 mm i.d. × 4.6 mm i.d. × 5 m PS C-18 chromatographic separation column was utilized. There is 0.2% triethylamine in the water portion of the mobile phase, which is a 70:30 combination of acetonitrile and water (pH 4). A 10-min trial run was possible with the setup at 272 nm, a flow rate of 0.8 mL/min, and a recycled injection capacity of 20 µL.

### 3.11. In Vivo Pharmacodynamic Studies

RZ’s anti-hypertensive and antianginal actions can be directly connected with the amount of medicine entering systemic circulation because it is prescribed for treating hypertension and angina. In other words, a rise in pharmacodynamic impact can indicate improved bioavailability. Since they are straightforward, quick, accessible, and simple to carry out in the lab, the DOCA salt (deoxycorticosterone acetate) model was chosen for examining the anti-hypertensive impact. The vasopressin-induced angina model was selected to research the antianginal effect in rats [52].

#### 3.11.1. DOCA Salt Model

In uni-nephrectomized rats, hypertension was induced using DOCA salt.

#### 3.11.2. Uni-Nephrectomy

The intraperitoneal injection of ketamine (100 mg/kg) was used to anesthetize male Sprague-Dawley rats weighing 250–300 g. A flank incision was made to remove the left kidney. A suture was used to connect the ureter and renal arteries. The incision was stitched shut using a chromic, sterile, absorbable suture [53].

#### 3.11.3. Induction of Hypertension

The rats received subcutaneous injections of DOCA (20 mg/kg) in olive oil twice a week for four weeks. The 1% NaCl solution was used as drinking water in its place. After one week, the blood pressure began to increase, and after four weeks, it was between 160 and 180 mm Hg.

#### 3.11.4. Experimental Design

Treatment with a simple drug suspension, RZ-NLCs, and RZ-NLCG was started after a 28-day induction of hypertension. The creatures were separated into four groups, each with six rats. With no therapy, group one acted as the control group. Group 2 received the DOCA dose of 20 mg/kg for the 28-day hypertensive control group. Rats in groups three and four were hypertensive. They were given oral doses of 2.5 mg/kg of RZ-NLCs and topically applied amounts of RZ-NLCG.

#### 3.11.5. Measurement of Blood Pressure

Using a pressure transducer on an MP30 data collection system (BIOPAC Systems Inc., Goleta, CA, USA), the systolic blood pressure was measured with a noninvasive tail cuff, and a digital display panel was used. Initial BPs of the rats were recorded, and hypertension was induced by injecting 1% NaCl desoxycorticosterone acetate (DOCA) (20 mg/kg/week subcutaneously for two weeks). Later, rats with a minimum mean BP of 150 mmHg were selected. A tracheotomy was made, the trachea was cannulated, and intraperitoneal ketamine injections (100 mg/kg) were used to anesthetize the rats. A cannula was placed in the carotid artery. To prevent thrombus from occluding the cannula, the cannula was attached to a disposable tubing system that continuously infused 0.9% heparinized saline at a rate of 24 mL/h. To ensure a consistent flow into the arterial system, the infusion fluid was maintained under pressure. After a 20-min stabilization period, the systolic blood pressure was measured by attaching the carotid cannula to the pressure transducer [54]. 

#### 3.11.6. Vasopressin-Induced Angina Model

Following vasopressin administration, the ECG alterations (ST segment elevation) were taken into consideration as a metric to show the presence of myocardial ischemia, which may be caused by coronary vasoconstriction [55]. ST segment elevation was defined as the difference in ST segment amplitude following and immediately before vasopressin delivery [56]. Four groups of six male Sprague-Dawley rats, each weighing 250–300 g, were used. With no therapy, group one acted as the control group. Vasopressin, 1 IU/kg i.v., was used for group two. Rats in groups three and four were hypertensive and were given, respectively, oral doses of 2.5 mg/kg of RZ-NLCs and topically applied doses of RZ-NLCG. Ketamine (100 mg/kg) was injected intraperitoneally to anesthetize the rats. After the administration of vasopressin (1 IU/kg) i.v., an electrocardiogram (ECG) was captured using a Biopac system (BIOPAC, Goleta, CA, USA). Vasopressin was proven to elevate the ST segment after intravenous injection. One hour before the delivery of vasopressin, the formulation was administered topically and orally to the animals at a dose of 2.5 mg/kg of RZ-NLCs and RZNLCG. Following the intravenous administration of vasopressin, ECG changes were recorded at the appropriate intervals. 

#### 3.11.7. Histopathology 

The slices of tissues immobilized in formalin were stained with hematoxylin and eosin (H&E). Afterward, the pieces were soaked in paraffin and serially cut (3 mm thick) using a microtome (Leica RM 2125, Wetzlar, Germany). The microscope inspected the above slides, and images were captured. Illustrative area images were further analyzed through Image Pro Plus (Media Cyberneties, Rockville, MD, USA) image analysis software. For light microscopy research, at least four excised skin samples were examined from each group, and each microscopic slide was examined for at least ten fields. The photomicrographs were scrutinized and classified as per the skin’s alterations using scores by scaling as severe (+++), moderate (++), mild (+), and nil (−). All histopathological assessment was carried out under strict blinding by the concerned pathologist to the different research groups’ treatment assignments. Using a semi-quantitative scoring system, the blinded histopathological examination was accomplished under a light microscope (BX50, Olympus Corporation, Tokyo, Japan). The severity and extent of cardiac histological parameters were examined separately according to the three layers of the heart (Myonecrosis, Inflammatory cells, and Edema). The assessment of severity for each of the layers of the skin tissue was scaled from 5 to 30 to represent (1) no change, (2) mild, (3) moderate, and (4) severe [57,58].

### 3.12. Short-Term Stability Study

The Q1A stability testing technique proposed by the ICH was used to assess the robustness of the new RZ-NLC formulations. Six months were spent storing these freshly manufactured RZ-NLCs at 53 °C, 252 °C/60% RH, and 402 °C/75% RH. PS, PDI, ZP, and EE were measured at 0, 1, 3, and 6 months to assess the durability of the RZ-NLCs’ new formulation.

### 3.13. Statistical Analysis

One-way analysis of variance (ANOVA) and Tukey’s significant difference (HSD) test were used as post hoc procedures for comparisons. IBM’s Statistical Product and Service Suite (SPSS) was utilized to analyze the data (Version 22, Armonk, NY, USA). The data were presented as Mean S.D. at the *p* ≤ 0.05 significance level.

## 4. Conclusions

With the help of high-pressure homogenization, RZ-encapsulated NLCs were made into an effective transdermal formulation. In both in vitro drug release trials and Ex vivo research, the most efficient lipid carriers were found to be in a size range that allowed them to permeate the skin. The final product’s consistency, drug concentration, pH, and rheological characterization were enhanced by incorporating the nanolipid formulation into a gel matrix. Therefore, the nanoparticle size of the novel formulation is encouraging for its therapeutic potential. The NLC’s formulation included lipid carriers, which slowed the RZ’s release. In vivo pharmacokinetic studies show that RZ-NLCG has significantly higher relative bioavailability than commercial formulations. Therefore, RZ-NLCs and RZ-NLCG may be an appropriate alternative to the traditional formulation for treating angina pectoris, providing the same advantages with less drug administration and less frequent dosing.

## Figures and Tables

**Table 1:** Optimization of RZ-NLC formulation by Box-Behnken statistical design.

Std	X1	X2	X3	Y1	Y2	Y3	Y4
1	187.5	22.5	25	65.81 ± 2.3	264.3 ± 5.39	0.394 ± 0.043	−29.6 ± 1.26
2	187.5	22.5	25	65.81 ± 2.3	264.3 ± 5.39	0.394 ± 0.043	−29.6 ± 1.26
3	187.5	15	20	74.39 ± 4.9	215.4 ± 6.5	0.291 ± 0.012	−31.4 ± 1.05
4	187.5	22.5	25	65.81 ± 2.3	264.3 ± 5.39	0.394 ± 0.043	−29.6 ± 1.26
5	187.5	22.5	25	65.81 ± 2.3	264.3 ± 5.39	0.394 ± 0.043	−29.6 ± 1.26
6	225	22.5	20	82.34 ± 2.6	159.7 ± 6.4	0.218 ± 0.028	−35.4 ± 2.15
7	187.5	22.5	25	59.37 ± 2.3	264.3 ± 5.39	0.394 ± 0.043	−29.6 ± 1.26
8	225	30	25	88.39 ± 3.1	159.3 ± 3.1	0.168 ± 0.033	−38.7 ± 0.61
9	187.5	30	20	78.43 ± 2.4	173.4 ± 8.6	0.235 ± 0.152	−30.2 ± 0.98
10	187.5	30	30	80.19 ± 6.3	194.2 ± 3.33	0.198 ± 0.032	−39.4 ± 2.15
11	150	22.5	30	76.38 ± 1.8	182.3 ± 2.84	0.167 ± 0.051	−41.9 ± 1.38
12	187.5	15	30	82.16 ± 4.5	118.4 ± 5.94	0.135 ± 0.038	−39.4 ± 1.52
13	225	22.5	30	74.52 ± 1.9	182.9 ± 3.51	0.184 ± 0.015	−38.4 ± 0.68
14	225	15	25	69.31 ± 8.3	238.6 ± 6.25	0.326 ± 0.102	−35.2 ± 1.53
15	150	22.5	20	60.28 ± 6.4	215.3 ± 3.38	0.314 ± 0.038	−28.4 ± 0.75
16	150	15	25	76.18 ± 5.2	153.1 ± 5.13	0.158 ± 0.028	−36.4 ± 2.38
17	150	30	25	59.43 ± 7.3	294.8 ± 2.49	0.372 ± 0.125	−34.6 ± 3.24

**Table 2:** Point prediction assessed RZ-NLCs optimized with their observed and predicted values.

Optimized Formula	Optimized Concentration	Responses	Predicted Value	Experimental Value
Solid Lipid (X1)	187.5	EE (%)	89.749	88.39 ± 8.1
Liquid Lipid (X2)	15	PS (nm)	118.4	118.4 ± 5.94
Surfactant (X3)	30	PDI	0.1525	0.135 ± 0.038
		ZP (mV)	−34.13	−29.6 ± 1.26

**Table 3:** Evaluation criteria for the RZ-NLCG optimized for various physicochemical and mechanical qualities.

Physicochemical Properties				Mechanical Properties.				
Viscosity (cps)	Spreadability (g-cm/s)	pH	Swelling Index	Firmness (g)	Toughness	Consistency (g/s)	Cohesiveness (g)	Index of Viscosity (g.s)
38513 ± 0.59	16.84 ± 0.32	6.35 ± 0.26	3.642 ± 0.005	0.158 ± 0.01	0.492 ± 0.15	1.486 ± 0.23	−0.08 ± 0.58	−1.068 ± 0.21

**Table 4:** The pharmacokinetic absorption parameter of the transdermal formulation of Rz-NLCG compared to the oral version sold (Ranozex tablet).

Pharmacokinetics Parameter	Marketed	RZ-NLCG
Intercept	−0.033964629	−0.020708832
Slope	2.891306469	2.845944685
C0 (mcg/mL)	778.585782 ± 25.36	701.3659608 ± 13.47
K(h^−1^)	0.07822054 ± 0.02	0.04769244 ± 0.002
Dose (mg)	100	100
Vd (mL)	128.437999 ± 8.34	142.5789183 ± 6.48
Vd (L)	0.128438 ± 0.24	0.142578918 ± 0.002
t_1/2_ (h)	8.85956546 ± 0.05	14.53060491 ± 0.59
Clearance (L/h)	0.01004649 ± 0.02	0.006799936 ± 0.004
AUC_0-t_ (µg.h/mL)	779.585782 ± 13.24	702.3659608 ± 14.52
AUC_1-t_ (µg.h/mL)	8820.125 ± 16.58	11,155.465 ± 20.46
AUC_t-inf_ (µg.h/mL)	1437.72977 ± 10.34	3928.505246 ± 18.94
AUC_Total_ (µg.h/mL)	11,037.4406 ± 13.49	15,786.33621 ± 18.73
C_max_	1039.68 ± 5.48	986.52 ± 8.45
T_max_	2.05 ± 0.13	4.09 ± 0.48
Relative Bioavailability	-	1.64 ± 0.13

**Table 5:** Effect of RZ-NLCsG on histopathological changes.

Group	Myonecrosis	Inflammatory Cell	Edema
Control	*-*	*-*	*+*
Vasopressin	*+++*	*+++*	*+++*
RZ-NLCs	*++*	*+*	*+*
RZ-NLCsG	*-*	*-*	*-*

(-) Nil; (+) mild; (++) Moderate; (+++) Severe.

**Table 6:** RZ-NLCs’ PS, PDI, ZP, and drug content stability profiles under various storage conditions (n = 3).

Stability Parameters	0 month	1 month	3 months	6 months
Storage conditions (5 ± 3 °C)				
PS (nm)	118.4 ± 5.94	125.28 ± 2.31	129.32 ± 3.52	135.67 ± 5.94
EE (%)	88.39 ± 8.1	85.37 ± 1.05	82.46 ± 1.29	76.49 ± 0.95
PDI	0.118 ± 0.028	0.135 ± 0.025	0.139 ± 0.038	0.153 ± 0.051
ZP (mV)	−41.9 ± 1.38	−43.68 ± 0.29	−41.26 ± 0.83	−48.92 ± 0.61
Storage conditions (25 ± 2 °C/60 ± 5%RH)				
PS (nm)	118.4 ± 5.94	294.35 ± 18.23	457.22 ± 0.00	958.34 ± 0.00
EE (%)	88.39 ± 8.1	69.38 ± 5.64	NM	NM
PDI	0.118 ± 0.028	0.85 ± 0.35	NM	NM
ZP (mV)	−41.9 ± 1.38	−49.35 ± 2.35	NM	NM
Storage condition (40 ± 2 °C/75 ± 5% RH)				
PS (nm)	118.4 ± 5.94	356.49 ± 25.34	NM	NM
EE (%)	88.39 ± 8.1	59.43 ± 6.49	NM	NM
PDI	0.118 ± 0.028	0.94 ± 1.23	NM	NM
ZP (mV)	−41.9 ± 1.38	−45.32 ± 2.11	NM	NM

**Table 7:** Experimental settings and design parameters for a Box-Behnken study.

Parameter	Low (−1)	Medium (0)	High (+1)
Independent Variables			
Solid Lipid (X1)	4	6	8
Liquid Lipid (X2)	1	2.5	5
surfactant (X3)	1	5.5	10
Dependent variables			
Y1: Entrapment Efficiency (%)	59.37 ± 2.3	88.39 ± 8.1	
Y2: Vesicle Size (nm)	118.4 ± 5.94	294.8 ± 2.49	
Y3: polydispersity index	0.118 ± 0.028	0.372 ± 0.125	
Y4: zetapotential (mV)	−41.9 ± 1.38	−29.6 ± 1.26	

## Data Availability

All the relevant data has been provided in the manuscript itself.
